# Acoustoelectric Effect due to an In-Depth Inhomogeneous Conductivity Change in ZnO/Fused Silica Substrates

**DOI:** 10.3390/s24196399

**Published:** 2024-10-02

**Authors:** Cinzia Caliendo, Massimiliano Benetti, Domenico Cannatà, Farouk Laidoudi

**Affiliations:** 1 Institute for Photonics and Nanotechnologies, IFN-CNR, Via del Fosso del Cavaliere 100, 00133 Rome, Italy; 2 Institute for Microelectronics and Microsystems, IMM-CNR, Via del Fosso del Cavaliere 100, 00133 Rome, Italy; massimiliano.benetti@cnr.it (M.B.); domenico.cannata@cnr.it (D.C.); 3 Research Center in Industrial Technologies CRTI, P.O. Box 64 Cheraga, Algiers 16014, Algeria

**Keywords:** ZnO, piezoelectricity, UV light, Rayleigh wave, harmonic wave, acoustoelectric effect, high-power UV light

## Abstract

The acoustoelectric (AE) effect induced by the absorption of ultraviolet (UV) light at 365 nm in piezoelectric ZnO films was theoretically and experimentally studied. c-ZnO films 4.0 µm thick were grown by the RF reactive magnetron sputtering technique onto fused silica substrates at 200 °C. A surface acoustic wave (SAW) delay line was fabricated with two split-finger Al interdigital transducers (IDTs) photolithographically implemented onto the ZnO-free surface to excite and reveal the propagation of the fundamental Rayleigh wave and its third harmonic at about 39 and 104 MHz. A small area of a few square millimeters on the surface of the ZnO layer, in between the two IDTs, was illuminated by UV light at different light power values (from about 10 mW up to 1.2 W) through the back surface of the SiO_2_ substrate, which is optically transparent. The UV absorption caused a change of the ZnO electrical conductivity, which in turn affected the velocity and insertion loss (IL) of the two waves. It was experimentally observed that the phase velocity of the fundamental and third harmonic waves decreased with an increase in the UV power, while the IL vs. UV power behavior differed at large UV power values: the Rayleigh wave underwent a single peak in attenuation, while its third harmonic underwent a further peak. A two-dimensional finite element study was performed to simulate the waves IL and phase velocity vs. the ZnO electrical conductivity, under the assumption that the ZnO layer conductivity undergoes an in-depth inhomogeneous change according to an exponential decay law, with a penetration depth of 325 nm. The theoretical results predicted single- and double-peak IL behavior for the fundamental and harmonic wave due to volume conductivity changes, as opposed to the AE effect induced by surface conductivity changes for which a single-peak IL behavior is expected. The phenomena predicted by the theoretical models were confirmed by the experimental results.

## 1. Introduction

Rayleigh waves are surface acoustic waves (SAWs) that travel along the surface of a solid medium. If the propagating medium is piezoelectric, the mechanical wave is associated with an electric potential wave Φ, having the same temporal and spatial periodicity as the SAW. The wave energy is mostly trapped onto the surface and undergoes an exponential decay in the direction normal to the surface. As a result, the SAW characteristics (i.e., the phase velocity and propagation loss) are highly sensitive to surface perturbations (such as a change of the temperature, pressure, surface mass density, humidity, or gas concentration, to cite just a few), and a huge number of papers related to SAW sensors is reported in the literature [1,2,3]. SAW sensors based on the acoustoelectric (AE) effect measure the changes in the electrical conductivity and dielectric properties of thin solid films or liquids through wave velocity and propagation loss measurements. The AE coupling between the traveling Φ and the charge carriers in the film (or in the adjacent liquid) slows the wave velocity and increases the propagation loss, which represent the SAW sensor response. Thanks to the contributions of many authors [4,5,6,7,8], the AE was able to account for much of the phenomenon regarding the SAW interactions with conduction electrons in piezoelectric semiconductors, as well as with ions in liquids. In reference [9], the first SAW sensor based on changes in the electrical conductivity of a thin surface film covering the wave propagation path was described; from there on, this topic has been the object of numerous theoretical and experimental studies. In reference [10], the change in SAW frequency caused by the interaction of the wave’s electric field and the free carriers in the thermally activated WO_3_ film is measured to sense H_2_S gas concentrations. In reference [11], the interaction between the electric field of the acoustic plate modes (APMs) in a quartz plate interacts with ions and dipoles in the surrounding liquid environment. Changes in the dielectric constant or conductivity of the liquid solution perturb the propagation velocity of the APMs, thus producing the sensor response. In all these cases, the AE effect is localized at the surface of the wave-propagating medium and causes a velocity and insertion loss (IL) change in the form of a single relaxation phenomenon: with increasing the surface conductivity, the acoustic wave velocity decreases monotonically and finally reaches a plateau, while the attenuation goes through a peak at a critical sheet conductivity [1,12].

Zinc oxide, other than being a piezoelectric material, is a semiconductor with a band gap of about 3.3 eV: if it absorbs UV light at 365 nm wavelength, electron–hole pairs are generated, thus resulting in a change of its electrical conductivity. Because of this property, a considerable interest has been collected around SAW devices based on thin ZnO polycrystalline layers as well as on ZnO nanostructures (such as nanoparticles, nanosheets, nanobelts, nanoflowers, …), due to their large surface-to-volume ratio, easy processing, and low final device cost, for fabrication of visible-blind UV detectors. Visible-blind SAW UV sensors based on GaN-films were studied in the early 2000s [13,14]: from there on, extensive research started that explores photoconductive piezoelectric nanostructures or thin layers, different types of substrates (such as Si, LiTaO_3_, LiNbO_3_, …), and different types of acoustic waves (Rayleigh waves, Lamb modes, Sezawa wave, …).

Previously, the authors have tested the UV-sensing behavior of the ZnO/SiO_2_-based SAW devices at low UV power values (up to 0.5 W); measures have been performed for both the fundamental and third harmonic wave and for both front and back illumination [15]. The experimental results showed that the sensitivity of the fundamental and third harmonic mode for back illumination (1044 and 2305 ppm/(mWcm^−2^) is larger than that for top illumination (1084 and 2488 ppm/(mWcm^−2^). Electrical measures [16] were performed onto a ZnO 400 nm thick in the 7.7 to 65.9 mW/cm^2^ range of incident UV power: the measures demonstrated that the current flowing between the IDTs fingers for front illumination is about twice the back illumination current, as expected due to the depth of voltage penetration, which increases up to the SiO_2_/ZnO interface. ZnO-based photoconductor sensitivity (4.3·10^−4^ (S/m)/W) is larger in front illumination than in back illumination (2.15·10^−4^ (S/m)/W), as expected, given the closer proximity of the metal electrodes to the UV-perturbed portion of the ZnO layer. Theoretical calculations of the SAW phase velocity and insertion loss variations due to the inhomogeneous electrical conductivity changes in depth of ZnO [17] have shown the dependence of the volume AE effect on the mode frequency, highlighting the complexity of the SAW UV sensor design in terms of the type of UV illumination (top or back), type of propagation wave (Rayleigh or Sezawa), electrical boundary condition, and ZnO layer thickness.

The present paper explores both theoretically and experimentally the AE effect in ZnO/fused silica-based SAW delay lines due to the in-depth inhomogeneous conductivity changes that take place inside the piezoelectric ZnO layer in response to the absorption of high-power UV light (from about 10 mW up to 1.2 W) at 365 nm from the back side of the structure. The wave acoustic path between the two IDTs (6.6 mm IDTs center-to-center distance) was illuminated by the UV light by means of a liquid light guide (3 mm diameter) 1 mm away from the SiO_2_ substrate. The phenomena predicted by the theoretical models do not account for the thermal effects due to the UV light adsorption in the ZnO layer. The observed changes of the SAWs attenuation and phase during the UV/dark/UV cycles were reversible and repeatable for the whole UV power range tested. The obtained results confirm that the ZnO-based SAW devices are suitable for the design and fabrication of low-cost UV light sensors that do not need extra masks for light filtering due to inherent ZnO spectral selectivity.

## 2. The AE Effect

When a SAW travels along a piezoelectric medium at velocity v, both mechanical and electric fields oscillate at the same frequency f=vλ and are mostly trapped at a depth of approximately one wavelength λ from the surface: as a result, a layer of electrical charges is formed at the piezoelectric medium surface. If the surface conductivity of the SAW propagating medium is perturbed (for example, when a conductive film is deposited onto the surface of the wave propagation path), the wave undergoes a decrease in velocity and an increase in the propagation loss. The perturbation theory [18,19,20] predicts the changes in the SAW velocity and attenuation Δα, which can be expressed by the following approximate formulas:

(1) $$\frac{\Delta v}{v_{0}}=-\frac{K^{2}}{2}\frac{\sigma_{s}^{2}}{\sigma_{s}^{2}+{\left(v_{0}\;c_{s}\right)}^{2}}$$

(2) $$\frac{\Delta \alpha }{k}=\frac{K^{2}}{2}\frac{v_{0}\;c_{s}\;\sigma_{s}}{\sigma_{s}^{2}+{\left(v_{0}\;c_{s}\right)}^{2}}$$

where *K*^2^ is the electromechanical coupling factor for the SAW device, σ_s_ = σ · *h* is the sheet conductivity of the layer, *h* and σ are the thickness and bulk conductivity of the layer, $∆v=v-v_{0}$, v, and $v_{0}$ are the perturbed and unperturbed SAW velocity,$\;\sigma_{c}=v_{0}c_{s}=v_{0}\left(\varepsilon_{0}+\varepsilon_{s}\right)$, and ε_0_ and ε_s_ are the dielectric permittivity of air and of the piezoelectric half-space. According to Equations (1) and (2), when the conductivity of the layer increases, the SAW velocity decreases, and the propagation loss increases. The plot of Equation (2) vs. σ shows a peak that occurs at the critical sheet conductivity σ_c_ (corresponding to the maximum rate of velocity decrease, the mid-point between the open and short circuit velocities). From Equation (2), one can see that α increases linearly for σ < σ_c_, and then it reaches a peak at σ = σ_c_ and subsequently decreases proportionally to σ^−1^ with further increasing conductivity. The critical conductivity $\sigma_{c}$ depends on the SAW wave vector k=ωv, and thus the attenuation peak of different harmonic modes is expected to fall at different σ_c_ values. The magnitude of the AE response to an external stimulus, $\frac{\Delta v}{v_{0}}$ and $\frac{\Delta \alpha }{k}$, is proportional to *K*^2^, and thus it is dependent on the type of the piezoelectric substrate, as well as on its crystallographic cut and wave propagation direction.

As previously reported, if the ZnO absorbs UV light at 365 nm wavelength, the generated electron–hole pairs interact with the SAW electric field, thus resulting in a phase shift and time delay across the SAW device. The UV light penetrates inside the ZnO following an exponential decrease with distance into the absorbing medium; as a result, an incremental change in the electrical conductivity of the ZnO upon illumination is expected to follow an exponentially decreasing behavior as well. The UV-induced AE effect cannot be defined as superficial but rather a volume-based effect because the light, penetrating the semiconductor layer, alters its electrical conductivity up to a certain thickness below the surface. As a result, the vertical distribution of the generated charge carriers (and thus of the electrical conductivity) is penetration depth dependent. If the loss due to surface reflection is ignored, Beer’s law describes the intensity of absorbed light as a function of distance y,

(3) $$I=I_{0}\cdot e^{-\beta y}$$

for δ=1β, with β being the absorption coefficient and δ the penetration depth defined as the distance after which light intensity has been reduced by a factor of *e*; *y* is the propagation direction of the light.

The coupling between electric field and strain in piezoelectric solids is accounted by the piezoelectric constitutive relations [20], which describe the interplay of stress *T*, strain *S*, and electric field *E* or potential Φ:

(4) $$T_{ij}=c_{ijkl}\cdot S_{kl}-e_{ijk}\cdot E_{k}$$

(5) $$D_{i}=\varepsilon_{ij}\cdot E_{j}+e_{ijk}\cdot S_{jk}$$

where *T_ij_* represents the stress vector (N/m^2^), *c_ijkl_* the elastic stiffness matrix (N/m^2^), *e_ijk_* the piezoelectric stress matrix (C/m^2^), Ek=−∂Φ∂xk the electric field vector (V/m), $S_{kl}=\frac{1}{2}\cdot \left(\frac{\partial u_{k}}{\partial x_{l}}-\frac{\partial u_{l}}{\partial x_{k}}\right)$ the strain component, *u*_*k*_ the mechanical displacement component along the Cartesian axis *x*_*k*_ (x_1_ = *x*, x_2_ = *y*, x_3_ = *z*), $D_{i}$ the electrical displacement (C/m^2^), $\varepsilon_{ij}^{\prime }=\varepsilon_{ij}-j\cdot \sigma /\omega$, $\varepsilon_{ij}$ the permittivity matrix (F/m), and σ the electrical conductivity (S/m); ω = 2πf, $j=\sqrt{-1}$. The piezoelectrically stiffened elastic constants of the photoconductive ZnO become complex as follows:

(6) cijstiff=cij+eik·ekjεii−jσω=cij+eik·ekjεii1+σ2ω2εij2+jeik·ekjσωεij21+σ2ω2εij2

Thus, the SAW velocity becomes complex. The SAW velocity and attenuation changes, $\frac{\Delta v}{v_{0}}$ and $\frac{\Delta \alpha }{k}$, due to the volume AE effect can be derived by using a finite element method, as described in the following paragraphs.

## 3. Simulation Methodology and Results: Eigenfrequency and Frequency Domain Study

**Figure 1:**
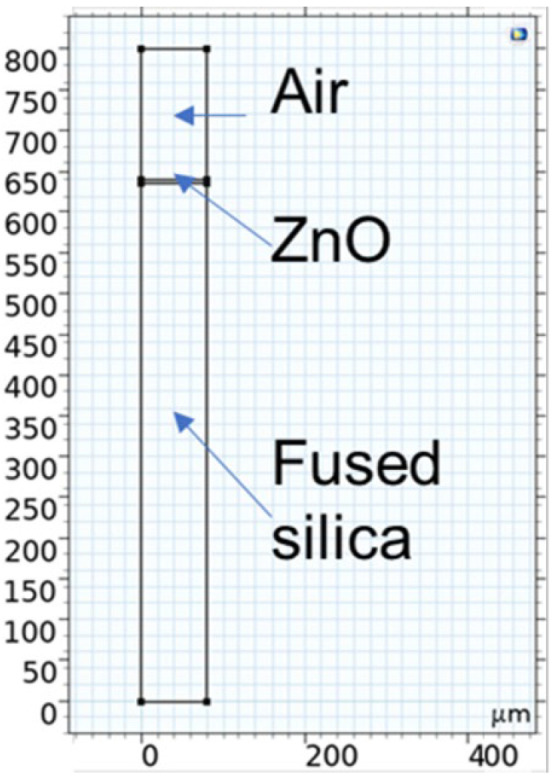
The unit cell, one wavelength wide (λ = 80 µm), showing the three domains.

Finite element simulations were performed using COMSOL Multiphysics 6.1 in the two-dimensional (2D) plane strain mode as no displacement is allowed transverse to the direction of wave propagation in c-ZnO/fused silica substrates. The model uses a piezoelectric Multiphysics coupling node with Solid Mechanics and Electrostatics interfaces. The unit cell (width equal to λ = 80 µm) has three domains, 10 boundaries, and 8 vertices: it consists in a two-wavelength thick air domain, a ZnO layer of thickness h_zno_ = 4 µm, and a fused silica domain (10·λ thick), as shown in Figure 1; *x* is the wave propagation direction and *y* is the depth. Both mechanic and electrical fields were considered for the SiO_2_ and ZnO layer (only electrical field for the air domain); periodic boundary conditions were applied to the left and right boundaries of the unit cell so reflections caused by the free edges could be ignored. The out of plane thickness of the whole domain was assumed to have the default depth given by the software (1 m).

**Figure 2:**
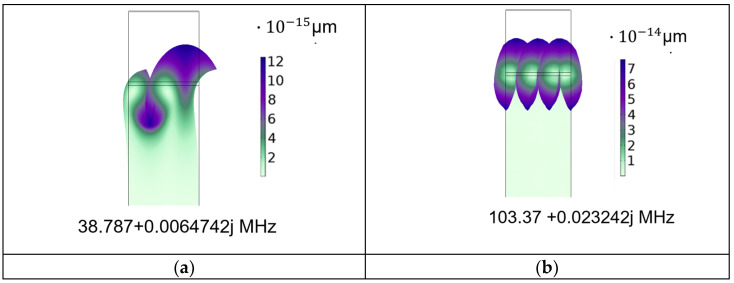
The solid displacement of (**a**) the fundamental Rayleigh wave and (**b**) of its third harmonic for σ = 10^−8^ S/m.

Eigenfrequency study was performed to calculate the symmetric and antisymmetric eigenmodes corresponding to the fundamental Rayleigh wave and to its third harmonic at 38.787 and 103.37 MHz: the frequency of the third harmonic wave is shorter than three times the frequency of the fundamental wave due to the phase velocity dispersion. Figure 2 shows, as an example, the zoom of the solid displacement of the two modes. The ZnO layer was assumed to be a single crystal with mechanical damping and dielectric loss equal to 0.002 and 0.01, and electrical conductivity σ = 10^−8^ S/m.

**Figure 3:**
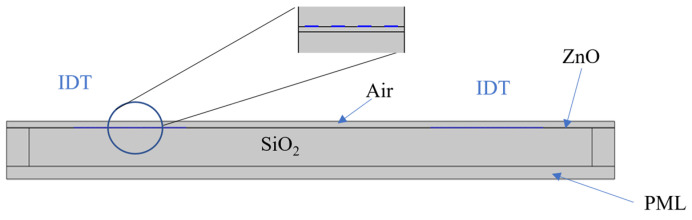
The 2D model (not in scale) of the PML/SiO_2_/ZnO/IDTs/Air structure; the inset shows a detail of the Al IDT fingers.

The frequency domain study was performed to obtain the amplitude and phase of the scattering parameter S_21_ (the transmission coefficient) of the SAW delay line. The 2D model studied reproduces the real dimensions of the device and includes the SiO_2_ domain (500 µm thick), a ZnO domain (4 µm thick), the air domain (with thickness equal to λ), and two IDTs located on the ZnO layer. Perfectly matched layers (PML) were assigned to the left, right, and bottom boundaries to eliminate the reflecting interference from the sides, as shown in Figure 3. Input and out Al IDTs were 80 fingers λ/8 wide (0.15 µm thick) with split finger configuration; the IDT center-to-center distance was 6600 µm. Two neighbor electrodes of the input and output IDTs had a terminated condition (power 1 W), while all the other fingers were grounded. The out-of-plane thickness of the whole domain was assumed to be equal to twice the fingers overlapping w of the IDTs implemented in the real devices tested (w = 1658 µm). Free triangular meshes were applied to the air domain; mapped quad meshes were applied to the SiO_2_ domain (100 elements, 75 element ratio onto the vertical boundaries, with reverse distribution); and custom meshes were applied to the ZnO domain (maximum and minimum element sizes of 0.7 and 0.265 µm onto the horizontal boundaries; maximum element size of 0.01 µm onto the vertical boundaries).

**Figure 4:**
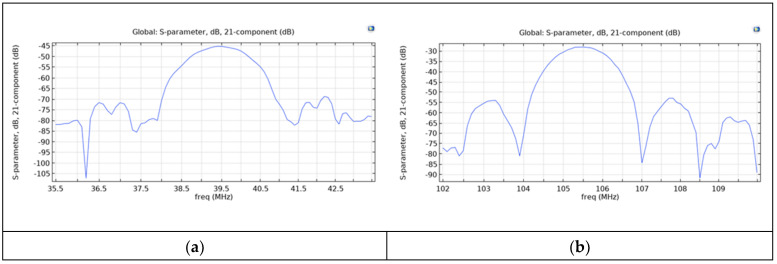
The S_21_ vs. frequency curve of (**a**) the fundamental Rayleigh wave and (**b**) of its third harmonic for $\sigma_{0}=10^{-8}$ S/m.

The scattering parameter S_21_ vs. frequency curves are shown in Figure 4a,b for both the fundamental and third harmonic waves for ZnO layer conductivity equal to 10^−8^ S/m (the delay line is supposed to be in dark).

By comparing the S_21_ vs. frequency curves of Figure 4, it can be noticed that, as expected, the passband minimum IL of the central frequency f_0_ of the Rayleigh wave was significantly larger than that of the third harmonic, due to the IDT split finger configuration that was optimized for the third harmonic wave [21].

A sweep parameter frequency domain study was performed to analyze the effect of the variable electrical conductivity of the ZnO layer (the changing parameter) onto the velocity and propagation loss of the two waves. It was assumed that the ZnO layer was illuminated from the back side (through the fused silica substrate, which is transparent to UV light at 365 nm wavelength, as was experimentally verified by the authors [16]) so that the ZnO would undergo a continuous exponential inhomogeneous conductivity profile that expires towards the ZnO/air interface. To try to reproduce the experimental conditions in which we operated, it was chosen to spatially limit the photoconductivity effect only inside a portion of the ZnO layer, located in between the two IDTs (i.e., only a small sub-domain of the ZnO layer had a variable permittivity, while the remaining part had fixed permittivity as it was supposed not to be illuminated by the UV light). Indeed, as will be described later, the tested devices were illuminated by UV light only in a limited area, located in the center between the two transducers, from the back side of the fused silica substrate, by means of an optical fiber connected to the UV laser led. The fiber had a diameter of 3 mm and was positioned very close (less than 1 mm away) to the back side of the device under test. The theoretical study was approached by assuming that inside the ZnO layer there was a central sub-domain with variable conductivity and hence variable permittivity according to the following expression:

(7) $$\varepsilon_{ij}^{\prime }=\varepsilon_{ij}-j\cdot \frac{\left[\sigma \left(x,y\right)\right]}{2\cdot \pi f_{0}}.$$

**Figure 5:**
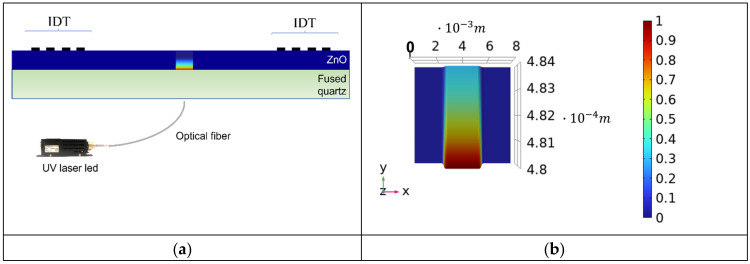
(**a**) The schematic of the device under UV illumination (not in scale). (**b**) The function $\frac{\sigma (x,\;y)}{\sigma_{0}}$ plotted over the ZnO layer’s thickness (the *y* direction) and width (*x* direction).

In Equation (8), the ZnO conductivity $\sigma \left(x,\;y\right)$ is described by the following discrete function of the spatial variables *x* and *y* (the wave propagation direction and the depth inside the ZnO layer):

(8) σx, y=σ0·Ax·e−(y−hSiO2)δUV

with $\sigma_{0}$ being the surface conductivity (the sweep parameter); $f_{0}$ the eigenfrequency of the mode, $\omega =2\cdot \pi \cdot f_{0}$; and $A\left(x\right)$ a rectangular function that has an aperture of 3 mm and a transition zone of 0.5 mm. $\sigma \left(x,\;y\right)$ decays exponentially towards the ZnO free surface, with a decay constant (the UV skin depth $\delta_{UV}$) equal to 325 nm; the $\delta_{UV}$ value of 325 nm was previously measured by the authors, as reported in reference [22]. Figure 5a shows the schematic of the device under UV illumination: the rainbow-colored ZnO layer reproduced the conductivity profile induced by UV illumination; in this picture, the UV power is assumed to be quite small so that the photoconductivity process does not involve the whole thickness of the ZnO layer. Figure 5b shows the plot of the function $\frac{\sigma (x,\;y)}{\sigma_{0}}$ in the *xy* plane, inside the ZnO layer: x extended across the width of the actual device (8 mm) and *y* over the thickness of the ZnO film (4 µm); the *z* axis was related to the magnitude of the plotted function.

**Figure 6:**
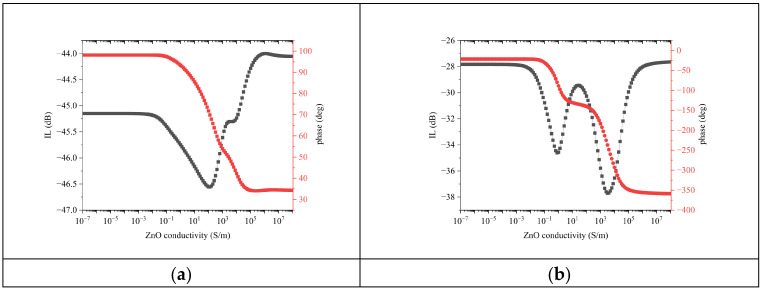
The S_21_dB (black colored) and phase (red colored) vs. conductivity curves of (**a**) the fundamental Rayleigh wave at 39.4 MHz and (**b**) of the third harmonic Rayleigh wave at 105.4 MHz.

A parametric sweep of the ZnO conductivity was performed to evaluate the AE effect on the velocity and IL of the Rayleigh waves: the ZnO electrical conductivity σ was varied from 10^−7^ up to 10^7^ S/m with 10^0.1^ step. The IL and phase at fixed frequency f_0_ (39.4 and 105.4 MHz for the fundamental and third harmonic waves) were extracted from the S_21_ vs. frequency curves and then plotted vs. σ, as shown in Figure 6a,b.

With increasing the *σ* of ZnO, the IL of the fundamental wave had a drop at about 100 S/m, then it decreased and reached a plateau at about 3981 S/m, and then it started to stabilize from about 10,000 S/m on. For σ ~2500 S/m, the IL was equal to its value in “dark”: any further increase in conductivity corresponded to a decrease in IL. When the conductivity varied from 10^−7^ to 10^7^, the ZnO film went from being insulating to conductive: for σ > 10^5^, the IL asymptotically stabilized at a value quite different from that corresponding to “insulating” piezoelectric ZnO onto SiO_2_. In this region, the ZnO layer was highly lossy, despite the lower IL with respect to the dark due to the suppression of the piezoelectricity in the “perturbed” ZnO layer. As a result, the S_21_ dB vs. σ curve shows a rapid drop of the IL since the wave launched by the IDT was unable to be revealed by the receiving IDT without a huge loss.

With increasing σ, the IL of the third harmonic wave worsened and reached a drop at about 0.79 S/m, and then it decreased up to a peak at 25 S/m, and finally it restarted to increases and reached a second drop at 3163 S/m. As in the case of the fundamental SAW, when the conductivity varied from 10^−7^ to 10^7^, the IL of the third harmonic asymptotically reached a value lower than that corresponding to “insulating” ZnO. The two peaks of the IL vs. σ curve of Figure 6b were separated by about four orders of magnitude and were clearly visible due to the logarithmic scale of the abscissa. If plotted in linear scale, the second peak appears as very broad and hardly reaching as it corresponds to an extremely high conductivity value.

The S_21_ phase of the fundamental SAW decreased monotonically with increasing σ, except for a rapid slope change at about 3981 S/m (the plateau of the IL curve), and then it started again to decrease and finally stabilized. The total phase drop was about 60 deg for the whole σ change.

The S_21_ phase vs. σ curve of the third harmonic SAW shows a behavior like that of the fundamental wave, but the slope’ change was more marked (a double steplike change of the phase takes place). The logarithmic scale of the abscissa made very clear the existence of both the double attenuation peak and the intermediate phase plateau. The total phase shift for the whole σ change (about 330 deg) was much larger than that of the fundamental SAW.

**Figure 7:**
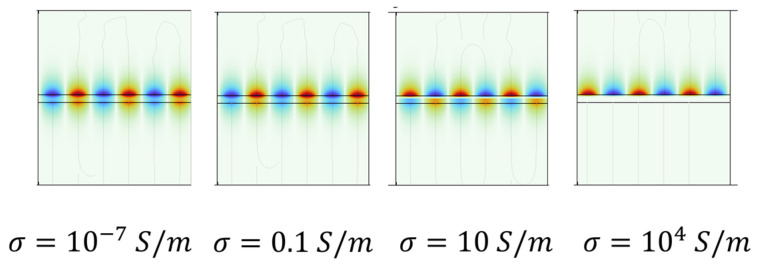
The electric potential profile of the third harmonic wave for σ = 10^−7^, 0.1, 10, and 10^4^ S/m (the pictures refer to the unit cell and were obtained by the eigenfrequency sweep parameter study).

Figure 7 shows, as an example, the electric potential Φ profile in the air/ZnO/SiO_2_ domains of the third harmonic wave, for some σ values (10^−7^, 0.1, 10, and 10^3^ S/m); the pictures refer to the unit cell with four split IDT electrodes.

For low σ, the electric potential was distributed inside the whole ZnO layer and had an exponential decreasing tail both in the air and in the SiO_2_ substrate. With increasing σ, the ZnO/SiO_2_ interface became conductive, and Φ was mostly concentrated inside an ever-smaller piezoelectric layer covering the portion of the ZnO that is involved in the photoconductivity process and has an exponential tail in air.

The presently adopted theoretical model does not consider any temperature-related effects that take place during the UV adsorption: precise modelling of the thermal process is intended for the purpose of further analysis to be performed soon.

## 4. Experimental Section

### 4.1. Materials and Measures

**Figure 8:**
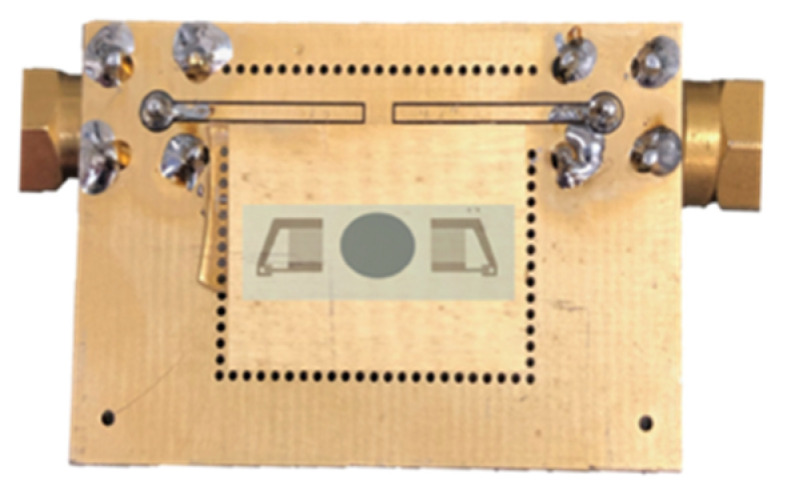
The SAW delay line mounted onto the PCB, which has a round hole to allow the back side illumination of the device.

ZnO thin films were grown onto fused silica substrates by an RF reactive magnetron sputtering technique equipped with a 4″ diameter high-purity (99.999%) zinc target by following a procedure outlined in references [23,24]. Conventional photolithographic and lift-off techniques were employed to pattern the IDTs onto an Al layer (1500 Å thick) grown onto the ZnO film by the RF sputtering technique from a high-purity Al target in Ar atmosphere: each IDT consisted of 80 electrodes with a periodicity of 80 μm; the acoustic aperture was equal to 1568 μm, and the IDT center-to-center distance was equal to 6600 μm. The SAW device was mounted onto a printed circuit board (PCB) with an epoxy adhesive and was electrically connected to the PCB using aluminum wire bonding. The PCB had a round hole to allow the illumination of the back side of the device, as shown in Figure 8.

**Figure 9:**
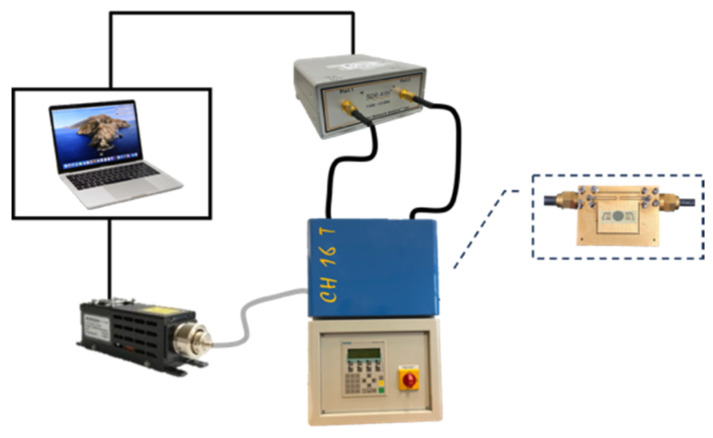
A schematic of the measured set-up including the climatic chamber that contains the SAW device illuminated by the UV high-power LED through the optical fiber; both the VNA and the UV LED were remotely controlled by the PC.

The scattering parameter S_21_ of the Rayleigh wave and its third harmonic was measured in the frequency domain by using a vector network analyzer (DG8SAQ VNWA 3 from SDR-Kits, Melksham, UK) in the dark and under UV illumination. The UV radiation from a high-power LED (LEDMOD365.3500.HP from Omicron-Laserage Laserprodukte GmbH, Rodgau-Dudenhofen, Germany) at 365 nm was focused at the center of the acoustic propagation path in between the two IDTs by means of a liquid light guide recommended for high-radiation power (LEDMOD.HP.3MM.LLG, 3 mm diameter), 1.5 m in length. The output power provided by the UV led was controlled by the software (Omicron Control Center (OCC)—Control Software v3.12.27), which configures the LED for computer-independent operation via RS-232 and the USB 2.0 interface: the UV power set point can be changed by minimum steps of 0.1% of the maximum power (3.6 W). Double readings are available of both the setpoint and the power values measured at the output of the LED; the effective power values that hit the device surface undergo an attenuation of about 60% from the LED/fiber adaptor and by the liquid light guide. Figure 9 shows a schematic of the measured set-up. The UV light was focused onto the lower side of the ZnO layer through the optically transparent fused silica substrate; a round hole in the PCB behaved like a shadow mask, allowing the limiting of the illuminated area to the acoustic path far from the IDTs. The scattering parameter S_21_ of the sensor was measured using the network analyzer, which was connected to the PC for real-time acquisition. The SAW device and the fiber were located inside a climatic chamber with black walls to avoid possible interferences from the surrounding environment. The climatic chamber was off during the experiments, and it was used only to isolate the SAW device from environmental perturbations. The S_21_ phase and IL at constant frequency were monitored in time during the dark–UV light–dark cycles.

**Figure 10:**
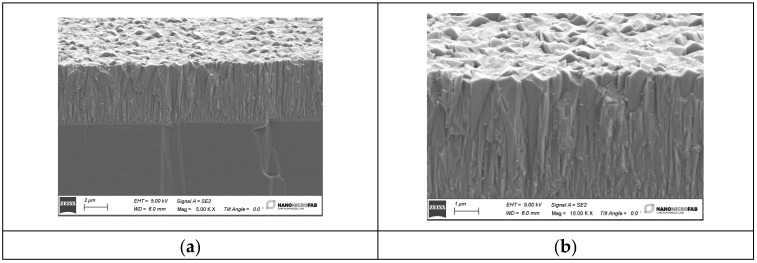
SEM images of the sputtered ZnO layers at different magnification (5.00 KX and 10.00 KX).

The sputtered ZnO films were c-axis oriented and showed a dense columnar structure: the surface micrographs of the ZnO thin films were recorded using a scanning electron microscope (SEM) Zeiss Evo MA10, as shown in Figure 10. The photos depict a columnar structure and refer to the ZnO film grown onto Si because the latter can be cleaved for cross-sectional imaging more conveniently than fused silica. The ZnO films were c-axis oriented, uniform, and highly adhesive to the substrate [21].

The measure of the optical transmission of the ZnO thin films was performed in the UV spectrum (from 200 to 450 nm) by using a Xe lamp and a spectrophotometer (Jobin Yvon Horiba Triax 190, Horiba, Edison, NJ, USA) in order to evaluate the UV penetration depth at different optical wavelengths. As a result, from the optical characterization described in reference [22], the absorption coefficient of our sputtered ZnO films was equal to 325 nm at an optical wavelength of 365 nm.

Current-voltage (I-V) measurements were carried out in the dark for the electrical characterization of the ZnO layers. One pad of the IDT was connected to the ground and the other pad to a DC voltage generator; the DC-positive bias voltage V was brought from 0 to 1 V with steps of 0.1 V (with 30 s time interval), and the current I flowing between the fingers of the split IDT was measured. The I-V curve displayed an ohmic behavior for the voltage range tested, and the resistivity was about 0.25 MΩ·cm.

### 4.2. Acoustic Measurements in the Dark

**Figure 11:**
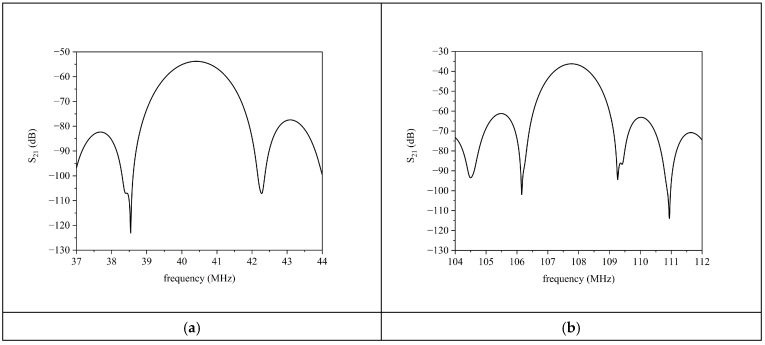
The S_21_ vs. frequency curve in dark for (**a**) the fundamental SAW and (**b**) the third harmonic wave.

The scattering parameter S_12_ of the SAW delay line implemented onto the ZnO layer surface was firstly measured in the frequency domain in the dark to identify the fundamental and third harmonic Rayleigh wave working frequency $f_{0}$ corresponding to the maximum of the S_21_ curve. Figure 11a,b shows the S_21_ vs. frequency curves for the fundamental and third harmonic SAW (*f*″ = 40.6 MHz and 107.75 MHz) in the dark: the delay line responses were gated in the time domain by using the time domain gate function and converted back to frequency. The time gate function is essentially a filter useful to suppress undesired interference codes (such as triple transit, multiple reflections, and electromagnetic feedthrough between the two metal IDTs) that overlap the signal of interest. The passband minimum IL of the third harmonic was significantly lower than that of the fundamental wave (as well as the maximum return loss of the fundamental wave being lower than that of the third harmonic), in accordance with the split finger IDT configuration that enables coupling at the third harmonic more efficiently than at the fundamental mode.

### 4.3. Acoustic Measurements in UV

Preliminary experimental measures of the AE effect for the fundamental and third harmonic Rayleigh wave in ZnO/fused silica were reported by the authors in [16,22] for a UV power range much smaller than that presently studied (maximum power was 0.45 W). It was observed that (i) the IL increased by increasing the incident UV power for both front and back side illumination; (ii) the front illumination produced a sensor response smaller than that produced by the back side illumination. Presently the AE effect was measured in back-illuminated mode for the two waves for a quite large UV power range (up to 1.2 W).

In most SAW delay line-based sensors, the difference of the phase between input and output signals is evaluated in the presence of an external stimulus: as the phase difference can be measured very accurately, the sensitivity of the device under testing can be evaluated with good accuracy. The AE effect was monitored by measuring the S_21_ amplitude and the phase shifts between the two IDTs at constant frequency ($f_{meas}$, corresponding to the peak of the S_21_ frequency spectrum in the dark) for various UV power values. When the UV light is absorbed by the ZnO layer, the wave amplitude and velocity decrease, thus resulting in an attenuated output signal and a shifted phase toward higher values by a degree that is related to the incident UV power. A frequency $f_{meas}$ was selected in the vicinity of the lowest IL, in the dark; the VNA excitation frequency was held constant at $f_{meas}$, and the resulting S_21_ shift in phase ∆Φ and in IL were monitored during dark–UV light–dark cycles. The UV-induced wave velocity shift Δv is revealed by the phase shift ∆Φ according to the following relationship:

(9) $$∆\Phi =2\pi f_{meas}\frac{L}{v_{SAW}}\frac{\Delta v}{v_{SAW}}$$

where L is the IDT center-to-center distance (i.e., the wave path). Although the change of the phase Φ=2πL/λ and frequency $f_{meas}=v/\lambda$ should exhibit the same trend, this was not our case since $f_{meas}$ was affected by changes in both the SAW velocity and attenuation.

The transient responses of the S_21_ amplitude and phase were measured as follows: (i) repeatedly at the same UV power, to assess the reversibility and repeatability of the SAW response to UV over the entire available power range; (ii) at various UV power values in on–off conditions, to assess the sensor ability to discriminate large and small values of UV power; (iii) at various UV power values in continuously increasing and decreasing UV power, in order to assess the existence of the saturation condition or of any memory effect. All the measures were performed for UV light abruptly switched on and abruptly switched off.

#### 4.3.1. Fundamental Mode

**Figure 12:**
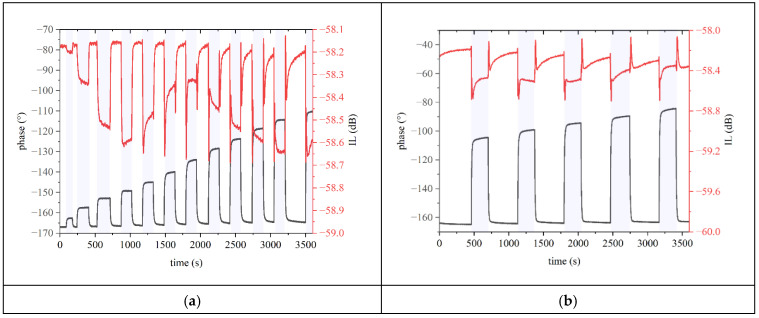

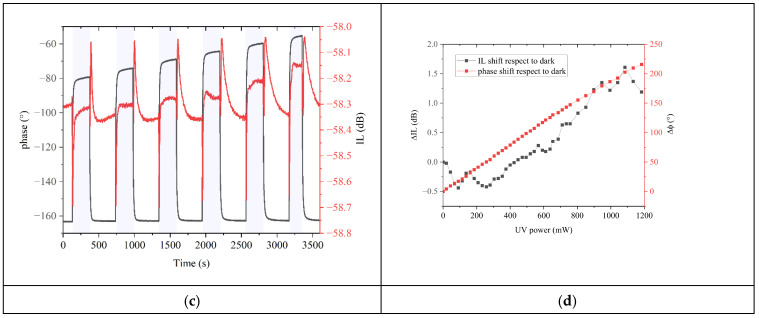
The time response (phase shift and IL vs. time curves) of the fundamental SAW during dark–UV light–dark cycles with UV power ranging (**a**) from 19 to 280 mW, (**b**) from 304 to 400 mW, and (**c**) from 423 to 543 mW, with a step of about 24 mW; the shadowed regions define the UV on condition. (**d**) The phase and IL shifts with respect to dark vs. UV power.

Figure 12 shows the time response (phase and IL vs. time curves) of the fundamental SAW during dark–UV light–dark cycles at some UV power values; red and black curves represent the IL and phase. Figure 12d shows the shift of the phase and IL with respect to dark vs. UV power curves.

Figure 12a shows the time response (phase shift and IL vs. time curves) of the fundamental SAW during twelve dark–UV light–dark cycles for UV power ranging from 19 to 280 mW, with steps of about 24 mW; times were around 2 min in the dark and around 2 min in UV, which seemed a time length sufficient for the phase to stabilize but not for the IL. Figure 12b,c show the IL and phase vs. time curves of the fundamental SAW during dark–UV light–dark cycles with UV power ranging from 304 to 400 mW, and from 423 to 543 mW, with a step of about 24 mW. The shadowed regions define the UV on condition. The sampling time was set to 0.1 s (ten measures per second). Figure 12d shows the phase and IL shifts vs. UV power; the shift was evaluated with respect to the dark. Under exposure to UV light at increasing power values up to 447 mW (Figure 12a,b and partially c), the IL went through a shift $\Delta IL={IL}_{UV}-{IL}_{dark}$ of increasing up to null magnitude; for larger UV power values, ΔIL became positive as the IL in UV ${IL}_{UV}$ was smaller than that in the dark ${IL}_{dark}$. The sensor response to UV light–dark–UV light cycles was tested several times to assess the measure’s repeatability.

**Figure 13:**
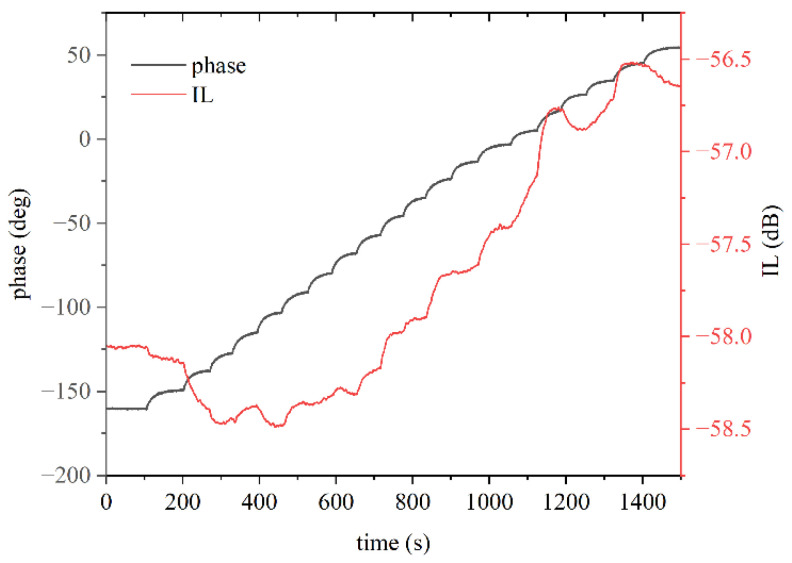
The phase and IL vs. time curves of the fundamental wave for UV power varying from 0 to 1.2 W with step of 5% of the maximum power.

Then, we moved on to illuminating the device with continuous and increasing power in steps of 5% of the maximum power. Figure 13 shows the time response (phase shift and IL vs. time curves) of the fundamental SAW for UV power increasing from 0 to 1.2 W.

#### 4.3.2. Third Harmonic Mode

**Figure 14:**
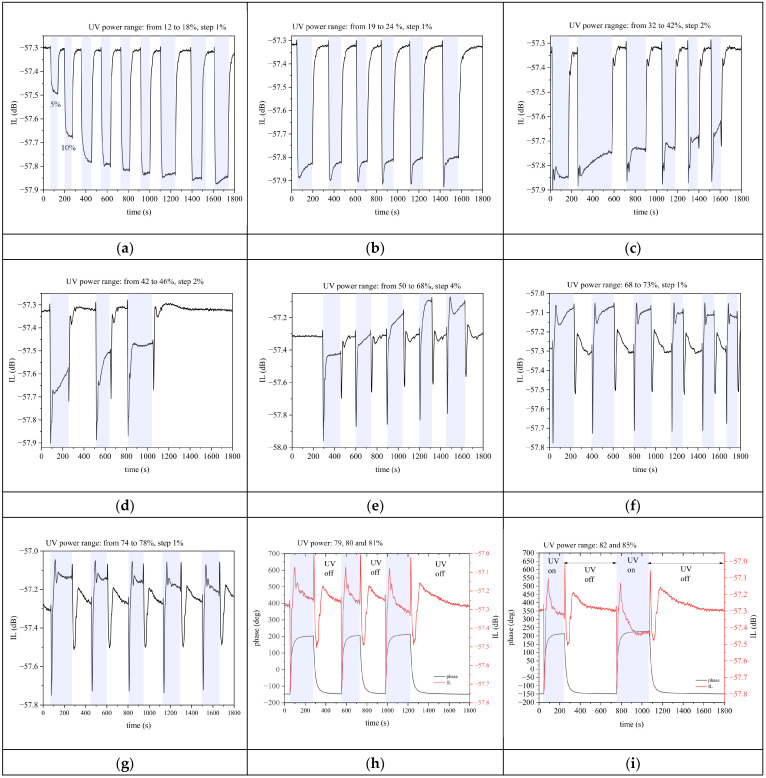
The IL vs. time curves of the third harmonic wave for UV power ranging (**a**) from 144 to 216, (**b**) from 228 to 288, (**c**) from 384 to 504, (**d**) from 504 to 552, (**e**) from 600 to 816, (**f**) from 816 to 876, and (**g**) from 888 to 936. The IL (red curve) and phase (black curve) vs. time curves for (**h**) 948, 960, and 972, and (**i**) for 984 and 1020 mW UV power.

Figure 14 shows the time response (IL vs. time curves) of the third harmonic wave during several dark–UV light–dark cycles for increasing UV power. The shadowed regions represent the UV on operating condition. The UV power ranged from 144 to 216 mW, from 228 to 288 mW, and from 384 to 504 mW in Figure 14a–c: at the beginning of the UV power cycles, the IL stabilized to a value IL_UV_ higher than in the dark (IL_dark_), but then the IL shift $\Delta IL={IL}_{UV}-{IL}_{dark}$ reduced its amplitude. When the UV power ranged from 504 to 552 mW, and from 600 to 816 mW (Figure 14d,e), the ΔIL progressively reduced its amplitude and even changed its sign (becomes positive), passing through zero. When the UV power ranged from 816 to 876 mW, and from 888 to 936 mW (Figure 14f,g), the ΔIL was positive but progressively decreased its amplitude and became null. When the UV power progressed from 948 to 1020 mW (Figure 14h,i), the ΔIL became negative again with increasing amplitude. The phase vs. time curves were added only into the plots of Figure 14h,i for clarity reasons.

**Figure 15:**
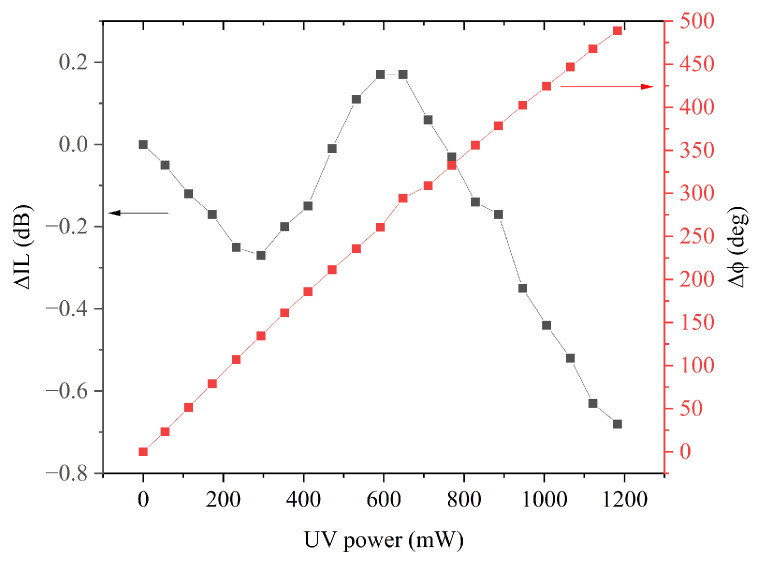
The IL and phase shifts with respect to dark vs. UV power for the third harmonic Rayleigh wave.

Figure 15 shows the IL and phase shifts with respect to dark vs. UV power curves (black and red colored) for the third harmonic Rayleigh wave; 292 mW was the UV power corresponding to the minimum IL shift.

**Figure 16:**
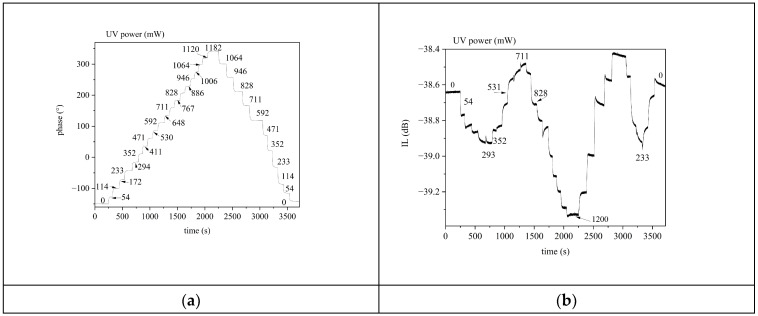
(**a**) The S_21_ phase at fixed frequency vs. time curve of the third harmonic Rayleigh wave and (**b**) the S_21_ dB at fixed frequency vs. time curve of the third harmonic wave, for power increasing from 0 to 1.2 mW, and then decreasing from 1.2 to 0 W.

Then, we moved on to illuminating the device with continuous and increasing power from 0 to 1200 mW in steps of 2% of the maximum power, and then with continuous and decreasing power from 1200 to 0 mW. Figure 16a,b shows the phase and IL vs. time curves of the third harmonic SAWs for UV power increasing from 0 to 1.2 W, and decreasing from 1.2 up to 0 W. The UV power values are written inside the two graphs. The UV power values of Figure 16b are the same as Figure 16a; only few UV power values are shown for clarity reasons.

## 5. Discussions

In the experimental set-up, the UV power source (which can provide the maximum output power of 3.6 W) was coupled to a flexible liquid optical guide that attenuated the UV light (1.2 W was the maximum output power at the optical fiber exit, and 3 mm was the fiber diameter). The fiber was positioned 1 mm away from the fused silica substrate (500 µm thick), and thus the optical radiation travelled a 1.5 mm length before reaching the ZnO layer surface. Reflections occurring at the air/SiO_2_ and SiO_2_/ZnO interfaces further reduced the optical power striking the central part (3 mm) of the acoustic path length (6 mm). Even after repeated measurement cycles, no damage to the surface of the ZnO layer nor any irreversible effects on the S_12_ were observed (after repeated cycles, the performance of the devices was unchanged compared to the dark). According to reference [25], heating might be partially responsible for the slower part of the sensor time response, while the fast part can be attributed to the screening of the SAW piezoelectric fields by the photoexcited carriers. During the UV/dark/UV cycles, the temperature changes of the ZnO/SiO_2_ substrate were not measured, and the samples were put inside a climatic chamber only to isolate the SAW devices from environmental perturbations.

The sensor sensitivities for the fundamental mode and its third harmonic (i.e., relative phase shift per unit UV power) were calculated as the slope of the linear fit of the experimental data shown in Figure 12d and Figure 15, respectively. The maximum phase shift of about 500° with a sensitivity of 0.14 deg/W was obtained for the third harmonic, while the sensitivity evaluated for the fundamental mode was 0.06 deg/W. For both the two waves, the phase increased monotonically with increasing conductivity of the ZnO layer: no appreciable changes of the slope were found within the limits of the accuracy of our measurements. The response and recovery times of the phase vs. UV power, for both the fundamental and third harmonic Rayleigh wave, were quite fast (around 10 and 13 s).

The behavior of the IL vs. UV power of the fundamental wave (described by Figure 12 and Figure 13) was quite close to the predictions obtained with the frequency domain study described in paragraph 2: when the increasing UV power was far below its maximum value, the IL of the fundamental wave saturated, as opposed to the IL of the third harmonic wave, which felt the effects induced by the whole UV power range onto the layer conductivity.

**Figure 17:**
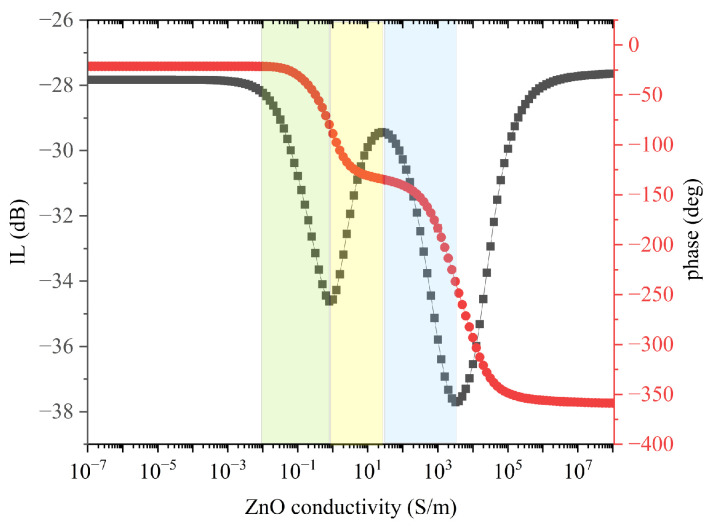
The IL vs. ZnO conductivity curve of the third harmonic wave with three colored areas superimposed on the curve. The green and yellow areas mark the conductivity range corresponding to an increase and a decrease in IL, respectively, while the blue area marks a second increase in IL below the ${IL}_{dark}$.

The IL of the third harmonic wave underwent a trend vs. the UV power similar to the theoretical IL vs. σ curve reported in Figure 6b, which is here re-shown for convenience (in Figure 17), but with some small modifications: three colored areas were superimposed on the IL and phase curves (black and red colored) vs. the ZnO conductivity to distinguish three different behaviors of the IL as the power of the UV light source variation. The three colored areas empirically delimit three ranges of electrical conductivity for which we currently have no evidence since the IL was measured as a function of the power of the UV LED and not of the electrical conductivity of the ZnO film. In this case, the green and yellow areas mark an increase and a decrease in IL, respectively; the blue area marks a second increase in IL below its value in the dark, ${IL}_{dark}$.

In light of our experimental measures and theoretical calculation, we deduce that the sputtered ZnO films have a dark conductivity lower than the ZnO/SiO_2_ characteristic conductivity σ_c_, as confirmed by the fact that, under low UV power, the SAW attenuation increased quite linearly. We can reasonably assume that the conductivity of the ZnO film increases under UV illumination and approaches $\sigma_{c}$ (the green area).

When the UV led power was increased further (the yellow area), the conductivity of the ZnO exceeded $\sigma_{c}$ and the SAW attenuation stabilized to values smaller than those of the green region but still larger than in the dark.

If the UV led power is increased further (the blue area), the SAW attenuation again starts to increase. In the blue region, the ZnO film acted as a sort of double-material layer consisting of a conductive sub-layer (close to the ZnO/SiO_2_ interface illuminated by the UV light) and an insulating sub-layer (close to the ZnO/air interface): by increasing the UV power, the depth of the light–matter interaction increased and a quite deeper portion of the ZnO layer was illuminated. As a result, the ZnO layer acted as a sort of bilayer consisting of a high-resistivity region (the portion not illuminated or poorly illuminated by UV light) and a region with increasing conductivity (and increasing thickness). When the UV power increased, the conductive sub-layer thickened and the upper insulating one thinned, hence affecting the electric potential distribution in the whole ZnO layer, as described by Figure 7.

Moreover, for equal ZnO layer thickness, harmonic waves with wavelengths shorter than that of the fundamental wave are expected to behave differently with respect to the absorbed UV light due to their different $\sigma_{c}$.

This empirical explanation of the observed IL vs. UV power trend is proposed to elucidate the double-peak sensing behavior of the SAW IL, even if, in the limit of the resolutions of our measures, we did not appreciate a change in the phase slope during the UV exposure. It is worth saying that our theoretical model is influenced by uncertainties in the material constants of both the substrate and the film. We are aware that the correct selection of the mechanical and thermal properties of the materials is a critical step that deeply affects the reliability of the numerical models that predict the SAW propagation characteristics with a reasonable accuracy. We can currently state that our theoretical results are reasonably in agreement with the average of the experimentally measured behaviors.

Actually, we do not know the correspondence between the power of the optical source that impacts the device surface and the resulting electrical conductivity of the ZnO layer: only the dark σ of the ZnO layers was measured from the current/voltage slope, as described in paragraph 3.1. During the exposure to UV light, the ZnO adsorbed the optical radiation, and the device underwent a temperature increase that was not monitored during the experiments performed; also, the theoretical calculations did not take into account any thermal effect. There is still much to be explored and studied on the subject, and works are planned to improve the theoretical and experimental study, as well as to measure the ZnO σ at high UV powers.

In SAW sensor applications, any changes of the environment’s parameters that alter the surface electrical conductivity of the wave-propagating medium can be detected by way of wave attenuation and phase changes. When the ZnO/SiO_2_-based SAW delay line is subjected to ultraviolet illumination, the incident light is absorbed by the semiconductor, and electron–hole pairs are generated, which interact with the electric field accompanying the propagating SAW, thus causing a downshift of the phase velocity and an upshift of the IL. This effect, however, corresponds to measurable sensing responses only for a limited range of the surface conductivity (about two orders of magnitude) around $\sigma_{c}$: if the perturbed film conductivity values fall outside this critical range, it would result in no significant device response. The $\sigma_{c}$ of a film/substrate structure is established by the electro-mechanical properties of the propagating medium (i.e., the product of the SAW velocity and the capacitance per unit length of the substrate), and thus it is expected to be different for the harmonic waves. The fabrication of SAW devices with different ZnO thicknesses and the excitation of multiple harmonic waves can be configured to produce a sensing platform with a wide range of critical conductivity values; each sensor can be tuned for detection in a specific conductivity range around the critical conductivity, allowing parallel analysis of the same stimulus with different sensitivities and degrees of accuracy.

ZnO-based SAW UV sensors offer advantages of low power consumption (due to their passive nature) and RF readout: they are suitable to construct zero-power and wireless UV detectors as opposed to conventional UV photodetectors that use either voltage or current as the output signal. ZnO-based SAW devices are suitable for low-cost, visible-blind UV light sensing as they do not need extra masks for light filtering due to the inherent ZnO spectral selectivity. Such sensors show long-term stability in harsh environments as the back UV illumination prevents the device surface from possible damage. The elevated surface area of the ZnO layer can generate large electron–hole pairs under UV light illumination, resulting in excellent device response.

The high structural quality of the ZnO layer is required to reduce the number of defects that otherwise serve as trap centers for the recombination of electrons and holes and reduce the charge carrier density, which decreases the device’s performance. The RF magnetron sputtering technique stands out for its technological ease: it is a well-established, high-deposition-rate, relatively low temperature (T = 200 °C) process that is compatible with standard microelectronic technology. All these features contribute to increasing the scientific world’s attention on ZnO/glass layered structures for UV-sensing applications.

There exists a wide literature focused on the characterization of SAW UV sensors based on ZnO films: the literature explores different types of substrates (such as LiNbO_3_, LiTaO_3_, quartz, glass, and Si) as well as different types of waves (such as Love waves, Lamb modes, fundamental and harmonic Rayleigh waves, and Sezawa waves [26,27,28,29,30]), as listed in Table 1. It is worth noting that most of the sensors have been tested at fixed UV power or over a small UV power range.

There also exists a wide literature focused on the characterization of the UV-sensing performance of SAW UV sensors based on ZnO nanostructures (such as nanorods, nanowires, nanosheets, nanobelts, nanoflowers, and nanoparticles, as particularly reported in the review paper of reference [32]). The high surface-to-volume ratio of such nanostructures is expected to help improve the performance of devices. Table 2 lists the sensitivities of a few UV sensors based on ZnO nanostructures for comparison.

It is not easy to make a comparison between the sensitivities of the various SAW UV sensors since the devices have been tested with different UV power values and with different experimental set-ups (such as front illumination of the whole device including the IDTs or of just one IDT).

The choice of the ZnO sensing layer, as opposed to the ZnO nanostructures, makes the sensor device perfectly reproducible and stable in time, even after several usage cycles.

The choice of the fused silica substrate, as opposed to Si or LiNbO_3_ substrates, makes the sensor intrinsically visible-blind and free of spurious interfering signals from the UV-sensitive substrate (such as LiNbO_3_) or from the non-insulating substrate (such as Si or SiO_2_ film onto Si substrate).

Finally, it is worth noting that, compared to the devices including the ZnO nanostructures, also the FEM study of the ZnO/SiO_2_-based SAW sensors benefits from the simplicity of the structure. The geometrical parameters of the nanostructures (such as cross-section, length, width, and thickness) are hardly reproducible, and moreover, their material properties can be significantly different with respect to their bulk counterparts. As a result, the complexity of the finite element model of ZnO nanostructures is overcome by the simplicity of the highly realistic model of the layer/substrate structure; the feasibility of a predictive model is a very important requirement since FEM simulations can help to predict the effects of any changes in the sensor design and evaluate its performance before proceeding to expensive prototyping and bench-top testing. We attributed the phase and IL shift to the interaction of the SAW with photoexcited carriers in the ZnO layer, but it is undeniable that the contribution of the latter should be accounted for in a more detailed analysis.

The examples listed in Table 1 and Table 2 are not exhaustive but help highlight the nature of the research, i.e., the test of the sensor’s performance for different substrates and acoustic wave types. Our research is curiosity-driven in nature as it explores the phenomenon to try to understand something and develop a realistic predictive model, which is a fundamental requirement to achieve an optimized design.

## 6. Conclusions

The AE effect induced by the absorption of UV light at 365 nm in ZnO/SiO_2_ substrates was studied for different light power values (from about 10 mW up to about 1.2 W) for both the fundamental Rayleigh wave and its third harmonic at about 39 and 104 MHz. The 2D-FEM study was performed to simulate the waves IL and phase velocity vs. the ZnO electrical conductivity, under the assumption that the ZnO layer conductivity undergoes an in-depth inhomogeneous change according to an exponential decay law, with a penetration depth of 325 nm. The theoretical results predict single- and double-peak IL behavior for the fundamental and harmonic wave due to volume conductivity changes, as opposed to the AE effect induced by surface conductivity changes for which a single-peak IL behavior is expected. The phenomena predicted by the theoretical models were confirmed by the experimental results. As opposed to most of the papers related to the ZnO-layer-based SAW UV sensors, the present paper focuses on the investigation of the sensing mechanism that takes place in a very simple structure, including an optically transparent and electrically insulating substrate (SiO_2_) covered by a photoconductive piezoelectric layer (ZnO). The UV-sensing mechanism of the ZnO/SiO_2_ sensors is activated by the ZnO photoconductivity property and is driven by the characteristics of the SAW propagation in bilayered structures.

Compared with other UV sensors, SAW sensors have many advantages, such as easy integration with integrated circuits, miniaturization, portability, remote wireless operation capability, and potentially zero power consumption. Moreover, multi-frequency ZnO/SiO_2_ structures are suitable for the development of multi-parameter sensing platforms for the parallel analysis in both gaseous and liquid environments [41].

We have provided an in-depth discussion on the UV-sensing mechanism through conceptual models, highlighting the importance of the two-material structure for efficient performance. Further work should be conducted to measure the temperature of the SAW device under UV illumination and to perform theoretical calculations that also take into account the thermal effects induced by the adsorbed UV light.

## Figures and Tables

**Table 1:** The sensitivity to some SAW UV sensors based on the propagation of different wave types.

Sensing Structure	Acoustic Wave	UV Power	Sensitivity	Ref.
ZnO/36° Y-cut LiTaO_3_	Love modes	570 μW cm^−2^	−2.5 dB without any significant frequency shift	[26]
ZnO/polyimide	Lamb modes	4.5 mW cm^−2^	55.8 ppm (mW cm^−2^)^−1^	[27]
ZnO/Si	Third harmonic Rayleigh wave	3 mWcm^−2^	400 kHz	[28]
AlScN(2 μm) on ultrathin flexible glass substrate	SAW	13.05 mWcm^−2^	1.71 KHz/(mW/cm^2^)	[29]
ZnO/Si	Sezawa	0.6 mWcm^−2^	25 kHz	[30]
ZnO/Al-foil (50 μm)	S_0_ Lamb mode	2–25 mWcm^−2^	53.7 ppm/(mW/cm^2^)	[31]

**Table 2:** The sensitivity to UV of some ZnO-based nanostructures.

Sensing Nanostructure	Substrate	Sensitivity at 365 nm	Ref.
Nanorods	ZnO nanorod film deposited on ST-cut quartz	~200 Hz at 6 μW cm^−2^	[33]
Nanowires	128° YX-LiNbO_3_	65 kHz under 150 μW/cm^2^	[34]
Nanowires	LiNbO_3_ substrate and Pt/Ti electrodes	0.26 ppm (mW/cm^2^)^−1^ sensitivity	[35]
Nanowires	Y-X cut 128° lithium niobate	9.53 ppm (mW/cm^2^)^−1^	[36]
Nanowires	128° YX-LiNbO_3_	65 kHz of the UV detector based on ZnO nanowire layer was observed under 150 μW/cm^2^ at 365 nm	[34]
Nanosheets	MoS_2_ nanosheets on IDTs/ZnO/glass	maximum frequency shift of ∼3.5 MHz under 365 nm UV 1.466 mW/cm^2^	[37]
Nanobelts	Bi_2_S_3_nanobelts on SAW delay-line sensor was fabricated on ST-cut quartz	upshift of 7 kHz within 1 s at625 nm visible light with a power intensity of 170 μW cm^−2^	[38]
Nanoparticles	ZnO NP/128° YX LiNbO_3_	3.34 ppm/μW cm^−2^	[39]
Nanoflowers	metal–semiconductor–metal (MSM) structure	6.34 × 10^4^ AW^−1^	[40]

## Data Availability

Data are contained within the article.
